# Комментарии к клиническим рекомендациям «Заболевания и состояния, связанные с дефицитом йода»

**DOI:** 10.14341/probl12823

**Published:** 2021-09-26

**Authors:** Г. А. Герасимов

**Affiliations:** Глобальная сеть по йоду

**Keywords:** йодный статус, йодированная соль, йодид калия, эндемический зоб, МКБ-10, нетоксический зоб, клинические ­рекомендации, Российская Федерация

## Abstract

В письме представлен анализ некоторых разделов клинических рекомендаций «Заболевания и состояния, связанные с дефицитом йода», опубликованных в №3 журнала «Проблемы эндокринологии» за 2021 г. В частности, обсуждаются разделы, посвященные кодированию заболеваний щитовидной железы по МКБ-10 в зависимости от йодного статуса населения отдельных субъектов Российской Федерации, а также вопросы диагностики и лечения, такие как «верификация» зоба, выявленного пальпацией, или назначение подавляющему большинству детей, подростков и взрослых людей препаратов калия йодида. Отдельно обсуждаются препятствия к эпидемиологической оценке йодного статуса населения при обследовании школьников в связи с введением в 2020 г. СанПиН, требующего обязательного использования йодированной соли для приготовления пищи в школьных столовых по всей стране.

## Abstract (en)

The Letter to Editor presents an analysis of some sections of the clinical guidelines «Diseases and conditions associated with iodine deficiency» published in No. 3 of the journal «Problems of Endocrinology» for 2021. In particular, the discussion deals with the coding of thyroid diseases according to ICD-10, depending on the iodine status of the population of constituent entities of the Russian Federation, as well as issues of diagnosis and treatment, such as «verification» of goiter detected by palpation, or treatment of the vast majority of children, adolescents and adults with potassium iodide. The obstacles to the epidemiological assessment of the iodine status of the population when examining schoolchildren are discussed separately, in connection with the introduction in 2020 of the new regulation, which requires the mandatory use of iodized salt for cooking in school canteens throughout the country.

Настоящая публикация, написанная в жанре «Письма в редакцию», ставит своей задачей анализ некоторых разделов клинических рекомендаций «Заболевания и состояния, связанные с дефицитом йода» (далее — Рекомендации), опубликованных в №3 журнала «Проблемы эндокринологии» за 2021 г. [1]. В целом эти Рекомендации являются очень полезным руководством для практических врачей и организаторов здравоохранения, и их необходимость и актуальность трудно переоценить. Вместе с тем некоторые разделы Рекомендаций, на мой взгляд, нуждаются в уточнениях и дополнениях, а другие — содержат спорные положения, требующие дополнительного обсуждения.

Поскольку в соответствии с современными требованиями [2] всем клиническим рекомендациям необходимо присваивать уровень убедительности (УУР) и уровень достоверности доказательств (УДД), было бы целесообразно привести в обновленной версии Рекомендаций расшифровку градаций шкалы оценки УУР и УДД, представленную ниже в таблицах 1 и 2.

**Table:** Таблица 1. Шкала оценки уровней убедительности рекомендаций (УУР) для методов профилактики, диагностики, лечения, медицинской реабилитации, в том числе основанных на использовании природных лечебных факторов (профилактических, диагностических, лечебных, реабилитационных вмешательств) [2]

УУР	Расшифровка
А	Сильная рекомендация (все рассматриваемые критерии эффективности (исходы) являются важными, все исследования имеют высокое или удовлетворительное методологическое качество, их выводы по интересующим исходам являются согласованными)
В	Условная рекомендация (не все рассматриваемые критерии эффективности (исходы) являются важными, не все исследования имеют высокое или удовлетворительное методологическое качество и/или их выводы по интересующим исходам не являются согласованными)
С	Слабая рекомендация (отсутствие доказательств надлежащего качества (все рассматриваемые критерии эффективности (исходы) являются неважными, все исследования имеют низкое методологическое качество и их выводы по интересующим исходам не являются согласованными)

**Table:** Таблица 2. Шкала оценки уровней достоверности доказательств (УДД) для методов профилактики, лечения, медицинской реабилитации, в том числе основанных на использовании природных лечебных факторов (профилактических, лечебных, реабилитационных вмешательств) [2]

УДД	Расшифровка
1.	Систематический обзор рандомизированных клинических исследований с применением метаанализа
2.	Отдельные рандомизированные клинические исследования и систематические обзоры исследований любого дизайна, за исключением рандомизированных клинических исследований, с применением метаанализа
3.	Нерандомизированные сравнительные исследования, в том числе когортные исследования
4.	Несравнительные исследования, описание клинического случая или серии случаев, исследование «случай-контроль»
5.	Имеется лишь обоснование механизма действия вмешательства (доклинические исследования) или мнение экспертов

Расшифровка УУР и УДД полезна также тем, что значительная их часть в обсуждаемых Рекомендациях имеет низкий уровень УУР (С) и УДД (4 и 5), что нужно принимать во внимание при их практическом применении.

Ряд пожеланий относится к разделу 1.4 «Особенности кодирования заболеваний или состояний (группы заболеваний и состояний) по Международной статистической классификации болезней и проблем, связанных со здоровьем».

К сожалению, этот раздел Рекомендаций включает в себя только список нозологий, относящихся к кодам Е01 и Е04 по МКБ, без каких-либо указаний по их практическому использованию. Учитывая, что Рекомендации посвящены заболеваниям, связанным с дефицитом йода, логично было бы сфокусировать внимание на болезнях щитовидной железы, связанных с йодной недостаточностью, и сходных состояниях (E01)1, к которым относятся [3]:

E01.0 Диффузный (эндемический) зоб, связанный с йодной недостаточностью;

E01.1 Многоузловой (эндемический) зоб, связанный с йодной недостаточностью. Узловой зоб, связанный с недостатком йода;

E01.2 Зоб (эндемический), связанный с йодной недостаточностью, неуточненный;

E01.8 Другие болезни щитовидной железы, связанные с йодной недостаточностью, и сходные состояния.

Однако, согласно «Аналитическому обзору по результатам мониторинга основных эпидемиологических характеристик йододефицитных заболеваний у населения Российской Федерации за период 2009–2015 гг.» [4], в России на начало 2016 г. было зарегистрировано на 30% больше пациентов с «другими формами нетоксического» зоба» (более миллиона), чем со всеми болезнями щитовидной железы, связанными с йодной недостаточностью, вместе взятыми (около 700 тыс.). При этом оставались непонятными критерии, которыми руководствовались российские врачи, относя часть диагнозов к группе заболеваний щитовидной железы, связанных с йодным дефицитом (Е01), а других — к, условно говоря, «спорадической» категории (Е04). Я не напрасно закавычил термин «спорадический», так как в современной научной литературе он практически не применяется, а МКБ-10 использует термины, перечисленные ниже [3].

E04.0 Нетоксический диффузный зоб.

E04.1 Нетоксический одноузловой зоб.

E04.2 Нетоксический многоузловой зоб.

E04.8 Другие уточненные формы нетоксического зоба.

E04.9 Нетоксический зоб неуточненный.

Хорошо известно, что на практике дифференцировать «спорадический» зоб от эндемического по клинико-лабораторным и инструментальным показателям практически невозможно2. Для этого используются не клинические, а эпидемиологические критерии: «спорадическим» зоб считается только в странах (или регионах внутри стран) с оптимальной йодной обеспеченностью, где эндемического зоба, связанного с йодной недостаточностью, не должно быть по определению [5].

Тут следует оговориться, что лишь очень небольшое число стран мира в принципе ведут систематический учет случаев нетоксического диффузного зоба (как «спорадического», так и эндемического). Вместо этого в США, например, национальные Центры по контролю за заболеваемостью (CDC) регулярно (примерно раз в 10 лет) исследуют концентрацию йода в моче (КЙМ) у репрезентативных групп населения, причем не по географическим регионам, а по этническим группам, а сведения размещают на своем официальном сайте [7]. Согласно опубликованным там данным, население США имеет адекватный йодный статус3. Сходная с российской система статистического учета заболеваний щитовидной железы существует только в скандинавских странах и некоторых бывших советских республиках.

Например, за последние 20 лет в Швеции не было зарегистрировано ни одного случая нетоксического зоба, связанного с йодной недостаточностью (E01), что не должно вызывать большого удивления: в этой стране с 1936 г. проводится йодирование соли, и уже много десятилетий тому назад был достигнут оптимальный йодный статус населения. А вот в Беларуси, устранившей дефицит йода около 20 лет тому назад, по-прежнему официально регистрируется заболеваемость эндемическим зобом (E01), хотя этой нозологии там уже быть не должно (медианная КЙМ как по всей стране, так и по отдельным областям находится в оптимальном диапазоне), а все новые случаи увеличения щитовидной железы должны в принципе регистрироваться как нетоксический зоб (E04) [5].

Однако, в отличие от Швеции и Беларуси, где действуют общенациональные эффективные программы йодной профилактики, в России ситуация с йодной обеспеченностью населения в существенной мере различается по отдельным регионам. После длительного перерыва Национальный медицинский исследовательский центр эндокринологии в 2020–2021 гг. стал вновь проводить мониторинг йодной обеспеченности: исследования были выполнены в Тыве, Крыму и Брянской области.

Наиболее обнадеживающие результаты были получены в Республике Тыва, в которой 25 лет тому назад имелись тяжелый йодный дефицит и даже случаи кретинизма. В 2020 г. 95,2% домохозяйств Тывы использовали йодированную соль, медианная КЙМ составила 153 мкг/л, а частота зоба у школьников — 7,7%. По данным медико-генетической службы республики, в 2019 г. при обследовании 6023 новорожденных только у 128 (2,1%) уровень тиреотропного гормона (ТТГ) превышал 5 мЕд/л, что тоже является свидетельством оптимального йодного статуса. Вместе с тем в 2017–2019 гг. на долю заболеваний щитовидной железы, связанных с дефицитом йода, приходилось 83,6% случаев [9]. Это противоречит здравому смыслу: если в Тыве устранен этиологический фактор (недостаточность йода в питании), то новые случаи зоба следует относить не к категории «связанных с йодной недостаточностью» (Е01), а к категории Е04.

Иная ситуация складывалась в Крыму и Брянской области. Так, в Крыму только 12,3% домохозяйств использовали йодированную соль, средняя частота распространенности зоба у детей по данным УЗИ щитовидной железы составляла 9,5%, а медианная КЙМ по районам полуострова варьировала от 78 до 98 мкг/л [10]. В Брянской области йодированную соль потребляли 17,8% домохозяйств, у 17% обследованных детей по данным УЗИ был выявлен диффузный зоб, частота которого по районам варьировала от 9,4 до 29%, а медианная КЙМ составляла 98,3 мкг/л4 [11].

Хотя йодный статус населения Брянской области не является адекватным, в 2020 г. у детей в возрасте 0–14 лет заболеваемость эндемическим зобом, связанным с йодной недостаточностью (Е01), была в почти в 20 раз ниже, чем «другими формами нетоксического зоба» (Е04): 1,0 и 19,1 случая на 1000 детей соответственно. У взрослых заболеваемость эндемическим зобом была ниже, чем «спорадическим» зобом, примерно в 6 раз [11]. Это нелогично: с учетом сохранения йодного дефицита все случаи зоба в этой области полагалось бы отнести к категории Е01.

Исходя из изложенных выше соображений, в раздел 1.4 Рекомендаций целесообразно внести требования по кодировке диагнозов (Е01 или Е04) в отдельных субъектах Российской Федерации в зависимости от статуса йодной обеспеченности целевых групп населения. К сожалению, данные о йодном статусе населения в большинстве регионов России либо отсутствуют, либо безнадежно устарели (ВОЗ рекомендует проводит такие оценки один раз в 3–5 лет). Вместе с тем внесение в Рекомендации требования по кодировке новых случаев болезней щитовидной железы в зависимости от йодного статуса населения могло бы подстегнуть интерес регионов России к такого рода исследованиям и проведению региональных профилактических программ с целью выхода из категории «эндемичных по зобу».

Помимо полевых исследований, йодный статус населения также может быть оценен по частоте увеличения концентрации ТТГ у новорожденных свыше 5 мЕд/л: при оптимальном йодном статусе этот показатель не должен превышать 3%. Учитывая высокий (более 90%) охват новорожденных неонатальным скринингом, который проводится во всех регионах России [10], статистический анализ баз данных с расчетом частоты увеличения концентрации неонатального ТТГ свыше 5 мЕд/л не должен быть затруднительной, а тем более — затратной процедурой.

В уточнениях также нуждается второй раздел Рекомендаций «Диагностика заболеваний или состояний, медицинские показания и противопоказания к применению методов диагностики».

Так, в подразделе 2.2.1 проведение ультразвукового исследования (УЗИ) щитовидной железы рекомендовано для верификации ее пальпаторного диффузного увеличения. В тексте Рекомендаций встречается многократное предупреждение, что «УЗИ щитовидной железы не рекомендуется как скрининговый тест»5. Но именно таковым это исследование и становится, если УЗИ будут назначать сотням тысяч ни в чем не повинных детей и взрослых, не говоря уже о возрастании бессмысленной нагрузки на диагностические службы. На мой взгляд, «верификация» диффузного нетоксического зоба в клинической практике с расчетом объема щитовидной железы по данным УЗИ и сопоставлением его с пороговыми нормативами является нецелесообразной. Как отмечают сами составители Рекомендаций, нормативы объема щитовидной железы (особенно у детей) созданы для эпидемиологических исследований, где превышение объема на десятую долю миллилитра превращает железу из условно нормальной в условно увеличенную. Но имеет ли это условное «увеличение» клиническое значение? Сомневаюсь. А вот при пальпаторном подозрении на наличие образования (узла) в железе УЗИ имеет важное значение, определяющее не только диагноз, но и тактику дальнейших действий [5].

В равной степени весьма сомнительна рекомендация (подраздел 2.3.1) исследовать уровень ТТГ в крови у пациентов с диффузным зобом для оценки функционального состояния щитовидной железы даже без наличия сколь либо значимых симптомов ее дисфункции. Авторы признают это сами, присваивая этой рекомендации УУР «С» и УДД 5 (самый низкий уровень убедительности и достоверности), а в комментарии признают, что «в районах с легким и умеренным йодным дефицитом гипотиреоз по причине йодного дефицита не встречается». Тогда зачем рекомендовать такое исследование?

Памятуя известное изречение М.Е. Салтыкова-Щедрина о том, что «строгость российских законов смягчается необязательностью их исполнения», я могу предположить, что в большинстве случаях практические врачи не будут в массовом порядке направлять пациентов с диффузным увеличением щитовидной железы на УЗИ и гормональный анализ. В романе Ф.М. Достоевского «Братья Карамазовы» один из героев в своем монологе произносит: «Широк, слишком широк человек. Я бы сузил». Вот и я тоже порекомендовал бы авторам при будущем пересмотре Рекомендаций сузить до разумных пределов показания к назначению УЗИ щитовидной железы и лабораторных диагностических исследований при диффузном нетоксическом зобе, особенно у детей и подростков.

Сложно согласиться и с рекомендацией по «консервативному лечению» диффузного зоба (подраздел 3.1.1), которая гласит: «На первом этапе лечения подавляющему (выделено мной) большинству детей, подростков и взрослых людей (моложе 40 лет) рекомендуется назначение калия йодида в дозе 100–200 мкг в день». В комментарии дается разъяснение, что «целью лечения ДНЗ является нормализация или уменьшение объема щитовидной железы». Впрочем, УУР (С) и УДД (5) этой рекомендации справедливо находятся на самом низком уровне.

Тут надо отметить, что процесс пополнения недостатка йода и других микронутриентов в питании в англоязычной научной литературе не удостаивается наименования «лечения», а описывается термином “supplementation”, что означает «добавление», «восполнение». И в этом есть определенный смысл. Ведь лечение обычно назначается врачом, тогда как добавки, содержащие йод, относятся к разновидности пищевых продуктов, именуемых БАД (биологически активные добавки)6. Восполнить недостаток йода можно и обогащенными пищевыми продуктами, главным из которых является йодированная соль.

Среднее ежедневное потребление соли на душу населения в России по крайней мере вдвое выше норматива в 5 г, рекомендованного ВОЗ, а большая часть соли поступает с промышленно обработанными пищевыми продуктами, в первую очередь хлебобулочными изделиями [12]. Вместе с тем, по данным недавней оценки, потребление соли непосредственно в домохозяйствах составляет примерно 3,5–4 г на человека в день [13]. В том случае, если соль йодирована согласно принятому в России нормативу (40 мг/кг) и с учетом потери 30% йода при кулинарной обработке, йодированная соль может реально обеспечить поступление около 100 мкг йода в день.

На мой взгляд, «подавляющему большинству» детей, подростков и взрослых людей любого возраста, особенно тем, у кого было выявлено диффузное увеличение щитовидной железы, вместо назначения таблеток йодида калия следует рекомендовать использовать дома только йодированную соль как для приготовления горячих блюд, так и присаливания холодных. Собственно говоря, именно это и советуют делать авторы Рекомендаций, правда, только после 6–12 мес приема йодных добавок. К сожалению, я не смог найти публикаций о степени приверженности пациентов приему таблеток йода в течение столь длительного срока. Однако, учитывая почти десятикратную разницу в цене, преимущество в комплаентности, скорее всего, будет на стороне йодированной соли7. Тем более что присутствие йодированной соли на кухне и в солонке на обеденном столе должно стать нормой в семье, а назначение йодных добавок может быть показано только тогда, когда эта «диетотерапия» не достигнет результата.

В завершение мне хотелось бы также прокомментировать раздел 7 Рекомендаций, посвященных эпидемиологической оценке распространенности йододефицитных заболеваний.

Во-первых, недавние рекомендации ЮНИСЕФ и Глобальной сети по йоду (ГСЙ) «Мониторинг программ йодирования соли и оценка статуса йодной обеспеченности населения» [14] изменили критерии оценки потребления йода населением, основанные на медианной КЙМ. В частности, адекватной теперь считается медианная КЙМ от 100 до 299 мкг/л (не мкг/сут, как ошибочно указано в тексте Рекомендаций). При этом осталась неизменной интерпретация медианной КЙМ более 300 мкг/л как избыточное потребление йода у детей школьного возраста. Эти изменения следует отразить в таблице 5 Рекомендаций.

Во-вторых, ЮНИСЕФ и ГСЙ указали [14], что исследования на базе школ не могут точно отражать йодный статус всего населения в странах, где развернуты широкомасштабные программы школьного питания с использованием йодированной соли. В Российской Федерации с начала 2020 г. действует СанПиН 2.4.5.2409-08 «Санитарно-эпидемиологические требования к организации питания обучающихся в общеобразовательных учреждениях, учреждениях начального и среднего профессионального образования», требующий обязательного использования йодированной соли для приготовления пищи в школьных столовых по всей стране. Таким образом, со школьными завтраками и обедами, приготовленными с йодированной солью, обследованные дети будут получать дополнительное количество йода и перестанут отражать йодный статус всего населения. Возможно, именно поэтому медианная КЙМ у школьников в Крыму и Брянской области была близка к нижней границе оптимального диапазона (от 100 до 300 мкг/л) в то время как только 12–17% домохозяйств использовали йодированную соль [10][11].

В такой ситуации ЮНИСЕФ и ГСЙ рекомендуют [14] проводить исследования йодного статуса на базе домашних хозяйств для устранения объективных ограничений исследований на базе школ. Такая модель исследования также может улучшить качество данных по охвату домохозяйств йодированной солью и йодному статусу таких групп населения, как беременные или женщины репродуктивного возраста. Вместе с тем эпидемиологические обследования домохозяйств требуют существенно больших затрат времени и средств по сравнению с исследованиями на базе школ. Вероятно, вопросам мониторинга программ йодирования соли в России следует посвятить отдельные методические указания, а в обсуждаемых здесь клинических Рекомендациях представить только общие принципы оценки йодного статуса населения.

В заключение хотел бы еще раз подчеркнуть важность и необходимость новых Рекомендаций, подготовленных на столь актуальную в России тему. Высказанные в этом письме замечания и пожелания относятся только к части обсуждаемых в них нозологий, в основном диффузному нетоксическому зобу (эндемическому или спорадическому), и могут быть учтены при их дальнейшем пересмотре.

1. Упомянутый в разделе 1.4 Рекомендаций код Е02 — субклинический гипотиреоз вследствие йодной недостаточности — был исключен из МКБ в 2016 г [3]. 2. Хотя КЙМ является хорошим индикатором йодного статуса в популяционных исследованиях, ее значение для оценки индивидуального йодного статуса ограничено высокой повседневной изменчивостью потребления и экскреции йода. У взрослых индивидуальные вариации КЙМ высоки при анализе как суточных, так и разовых проб мочи, поэтому для оценки индивидуального йодного статуса с точностью ±20% необходимо исследовать концентрацию йода примерно в 10 пробах мочи, собранных в разные дни [6]. 3. Строго говоря, даже в странах, где в целом у жителей нет дефицита йода, у отдельных групп населения, придерживающихся определенных диетических ограничений, например, веганов, имеется риск развития йодной недостаточности и йододефицитного зоба [8]. 4. Скорее всего, медианная КЙМ у школьников в описанных выше исследованиях может быть завышена и неточно отражать йодного статус всего населения из-за того, что с начала 2020 г. вступил в силу СанПиН 2.4.5.2409-08, требующий обязательного использования йодированной соли для приготовления пищи в школьных столовых по всей стране. Таким образом, для последующих исследований йодного статуса населения целесообразно выбирать другие целевые группы, например, женщин репродуктивного возраста или беременных. 5. Под «скрининговым тестом», видимо, понимаются массовые обследования здорового населения с использованием УЗИ, а также ситуации, когда УЗИ щитовидной железы проводится без всяких на то показаний в «нагрузку» к исследованиям других органов. 6. В России некоторые добавки, содержание физиологическую норму йода в форме йодида калия, могут быть зарегистрированы не как БАД, а как лекарственные средства, что не изменяет смысл их использования. 7. Быстрый поиск в интернете (сентябрь 2021 г.) показал, что цена 100 таблеток йодомарина-100 в Москве начинается от 150 руб., а цена 1 кг упаковки йодированной соли «Полесье» — от 16 рублей; этого количества соли будет достаточно для семьи из 3 человек примерно на 3 месяца.

## ДОПОЛНИТЕЛЬНАЯ ИНФОРМАЦИЯ

Источники финансирования. Работа выполнена по инициативе авторов без привлечения финансирования.

Конфликт интересов. Автор декларируют отсутствие явных и потенциальных конфликтов интересов, связанных с содержанием настоящей статьи.

Участие авторов. Автор одобрил финальную версию статьи ­перед публикацией, выразил согласие нести ответственность за все аспекты работы, подразумевающую надлежащее изучение и решение ­вопросов, связанных с точностью или добросовестностью любой ­части ­работы.
